# Supplementary material for: Spatial transcriptomics analysis of esophageal squamous precancerous lesions and their progression to esophageal cancer
Source: Nat Commun. 2023 Aug 8;14:4779. doi: 10.1038/s41467-023-40343-5 (PMC10409784; doi:10.1038/s41467-023-40343-5)
Supplement: Supplementary file 1 — Supplementary information [file 41467_2023_40343_MOESM1_ESM.pdf]

Supplementary information

Spatial transcriptomics analysis of esophageal squamous precancerous lesions and their progression to esophageal cancer

Xuejiao Liu[1,2], Simin Zhao[2,3,4], Keke Wang[2], Liting Zhou[1,2,4], Ming Jiang[2], Yunfeng Gao[2], Ran Yang[2], Shiwen Yan[1,2,4], Wen Zhang[1,2,4], Bingbing Lu[1,2,4], Feifei Liu[1,2,4], Ran Zhao[1,2], Wenting Liu[1,2], Zihan Zhang[1,2,4], Kangdong Liu[1,2,4,5,6], Xiang Li[1,2,4,5,6]*, Zigang Dong[1,2,4,5,6]*

[1]Department of Pathophysiology, School of Basic Medical Sciences, Zhengzhou University, Zhengzhou, Henan, China.

[2]China-US (Henan) Hormel Cancer Institute, Zhengzhou, Henan, China.

[3]Department of Pathology, Affiliated Cancer Hospital of Zhengzhou University, Zhengzhou, Henan, China

[4]Tianjian Laboratory of Advanced Biomedical Sciences, Institute of Advanced Biomedical Sciences, Zhengzhou University, Zhengzhou, Henan, China.

[5]The Collaborative Innovation Center of Henan Province for Cancer Chemoprevention, Zhengzhou, Henan, China.

[6]State Key Laboratory of Esophageal Cancer Prevention and Treatment, Zhengzhou University, Zhengzhou, Henan, China.

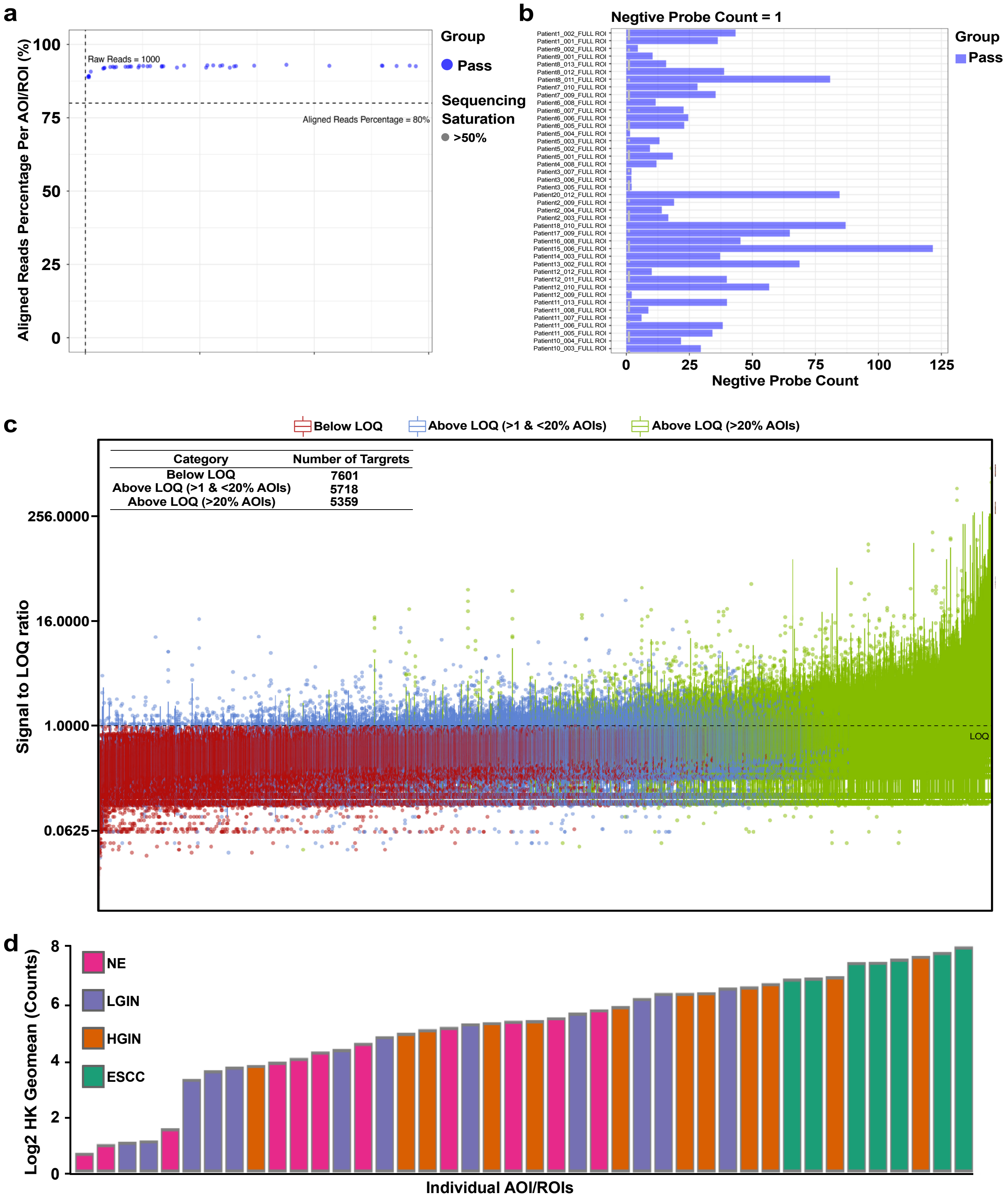

Supplementary Fig.1 Technical signal quality control of spatial whole-transcriptome atlas (WTA) analysis. a. Sequencing saturation is suggested over 50%. b. Technical background quality control of WTA analysis. c. The limit of quantitation (LOQ) showed a limit value for the confirmation target expression. d. The HouseKeeping Gene (HK) geomean value of each ROI. HK was used as normalization quality control.

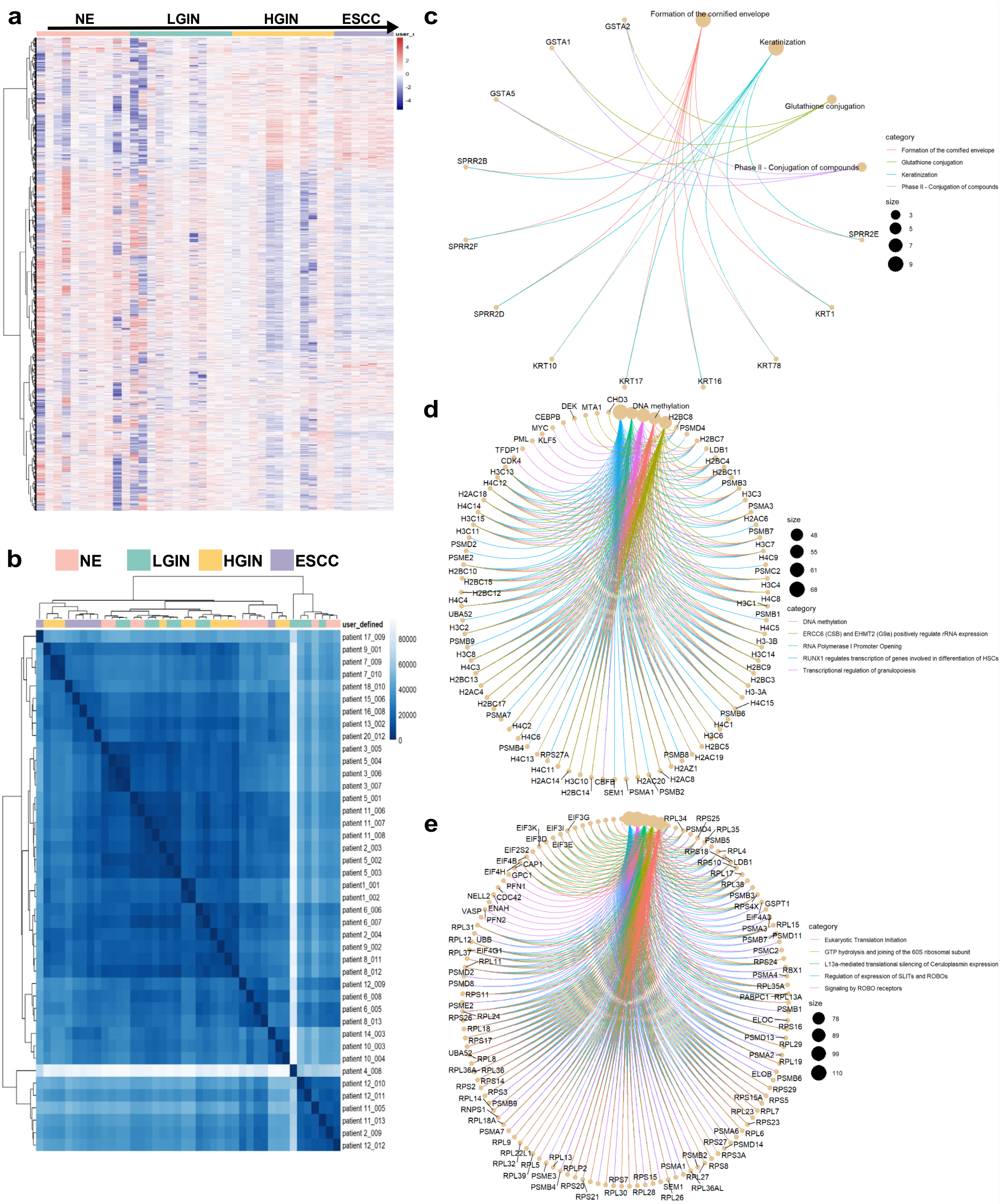

Supplementary Fig.2 Gene expression heatmap and related signaling pathways. a-b. The gene expression heatmap and cluster analysis of NE, LGIN, HGIN, and ESCC tissues. c. Signaling pathway change and the related genes involved in the signaling pathway of LGIN compared with NE. d. Related DEGs involved in the signaling pathway change of the HGIN group compared with NE. e. Signaling pathway change and the related DEGs involved in the signaling pathway of the ESCC group compared with the NE group.

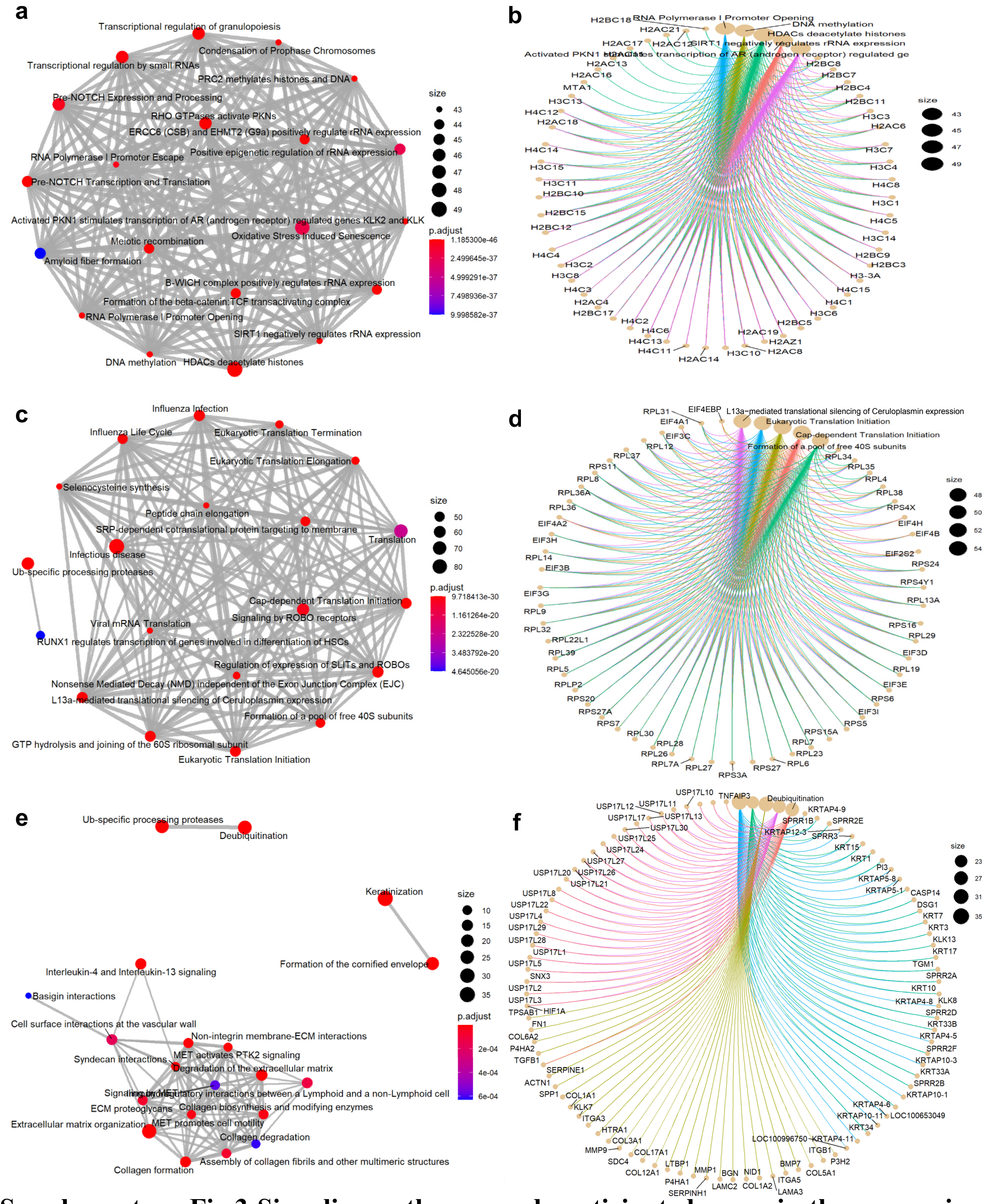

Supplementary Fig.3 Signaling pathways and participated genes in the comparison of HGIN and LGIN; ESCC and LGIN; ESCC and HGIN. a-b. The emapplot and related genes are involved in the signaling pathway of HGIN compared with LGIN. c-d. The emapplot and related genes involved in the signaling pathway in the comparison of ESCC and LGIN. e-f. The emapplot and related genes involved in the signaling pathway in the comparison of ESCC and HGIN.

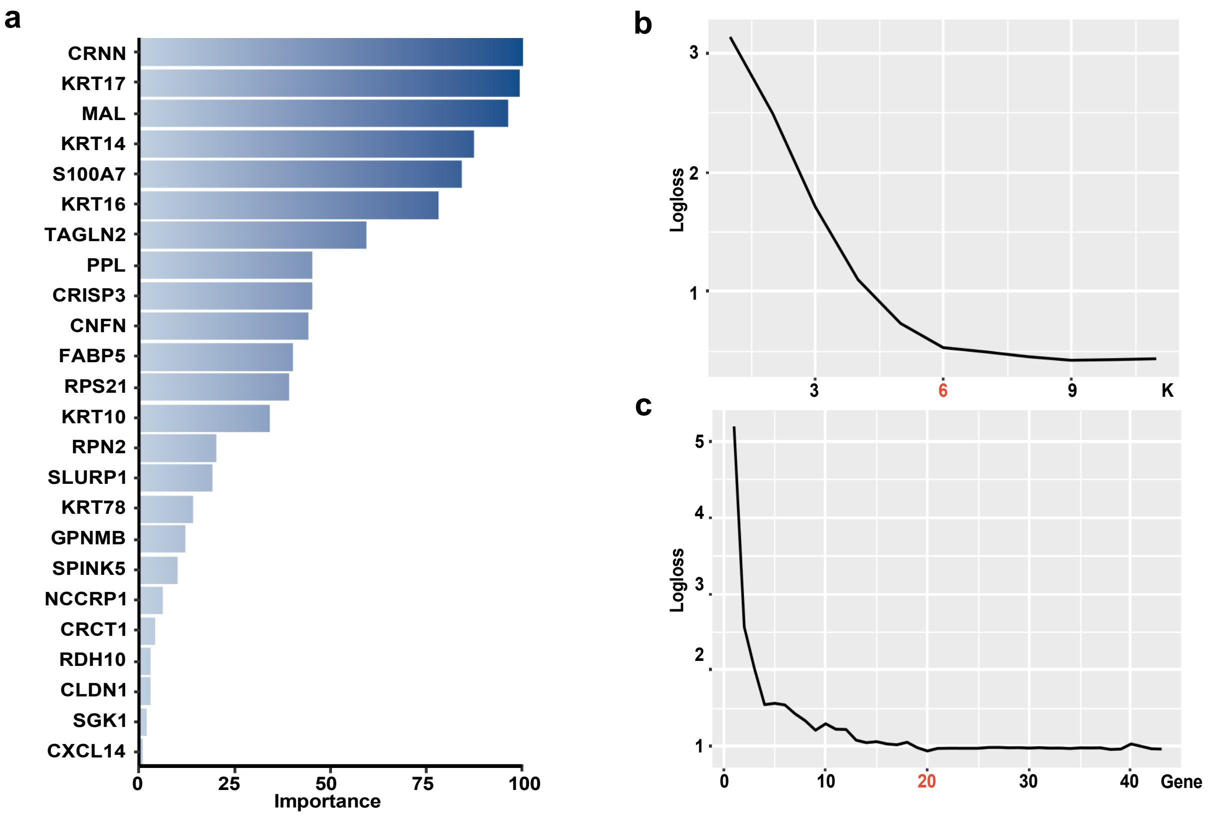

Supplementary Fig.4 Feature selection by machine learning models. a. The importance rank is analyzed by logre algorithm. b. K value selection by knn algorithm. c. Gene number selection by knn algorithm.

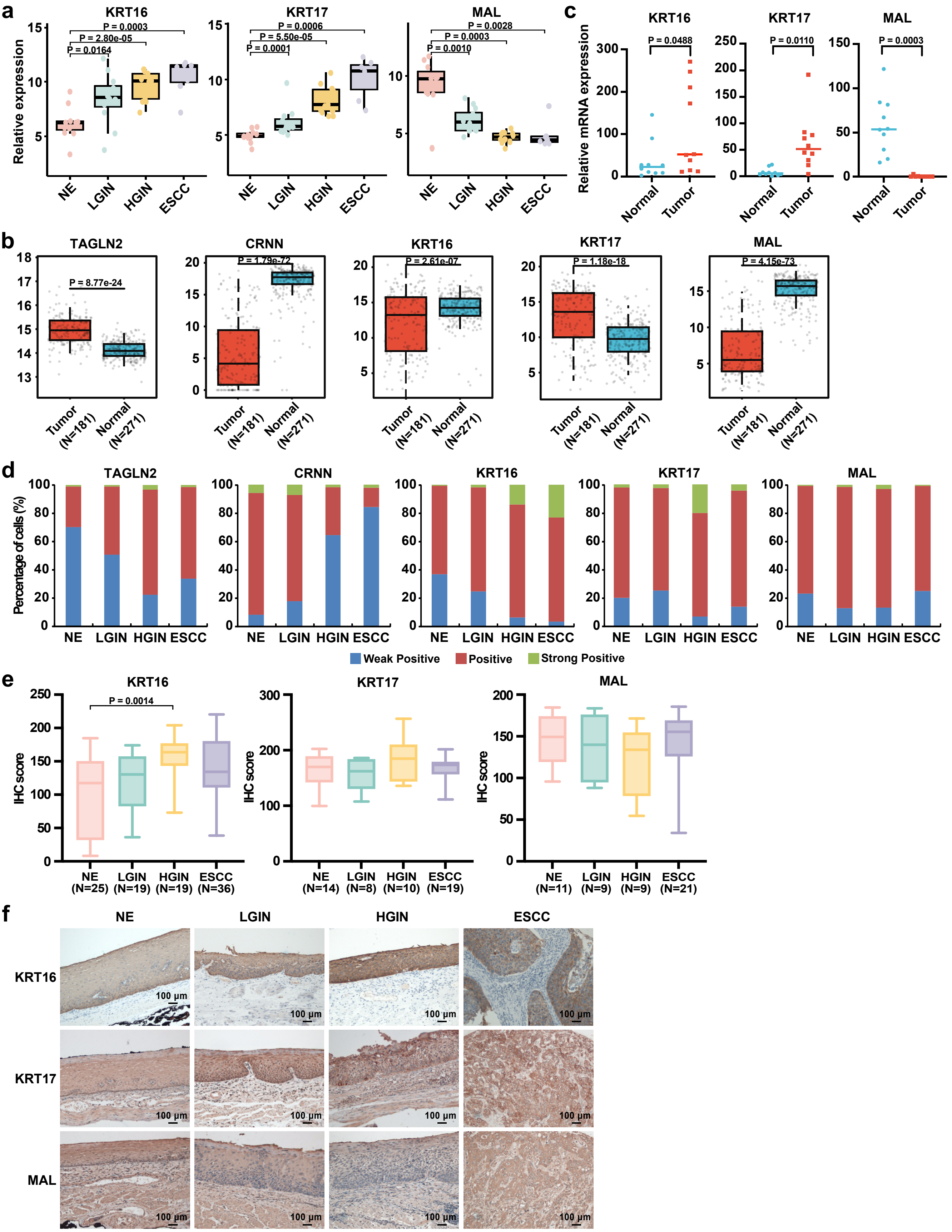

Supplementary Fig.5 Candidate genes expression in different pathological stages. a. Candidate genes *KRT16*, *KRT17*, and *MAL* expression in the four pathological stages by

spatial WTA analysis (Mann-Whitney U test). NE, LGIN, HGIN, and ESCC ROIs number (n = 11, 12, 12, and 7). b. *TAGLN2*, *KRT16*, *CRNN*, *KRT17*, and *MAL* gene expression in normal and esophageal cancer tissue from the TCGA database (two-tailed unpaired t-test). In the box plots (a, b), the boxplot shows the median (central line), upper and lower quartiles (box limits), and 1.5 × interquartile range (whiskers). c. Relative mRNA expression of candidate genes in paired normal (n = 10) and ESCC tissue (n = 10) biologically independent samples. The data was analyzed by a two-tailed paired t-test. d. Stacked histograms of weak positive, positive, and strong positive stained cells by IHC staining. e-f. Positive staining statistics and representative pictures of KRT16, KRT17, and MAL staining by IHC analysis (Scale bar, 100 μm). Kruskal-Wallis test and corrected by Dunn's test for multiple comparisons. The sample size is labeled in the figure. The boxplot shows the median (central line), upper and lower quartiles (box limits), and min to max range (whiskers). Source data are provided as a Source Data file.

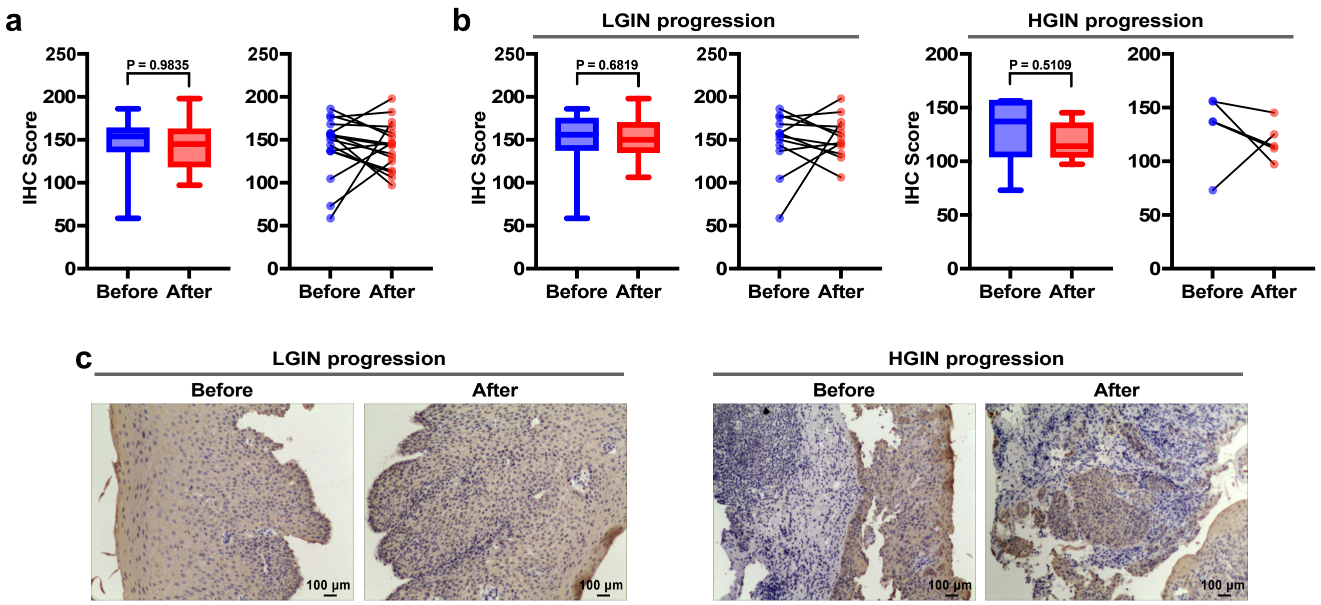

Supplementary Fig.6 Verification of the association between *KRT16* and ESCC progression. a. Analysis of the KRT16's IHC score was conducted on all paired progressed samples (including LGIN progression samples (n = 12) and HGIN progression samples (n = 5) biologically independent samples). b. Statistics of the IHC scores of KRT16 in paired LGIN progression (n = 12) and HGIN progression (n = 5) samples, respectively. c. Representative images of KRT16's IHC staining in paired LGIN progression and HGIN progression samples (Scale bar, 100 μm). In the box plots (a, b), the boxplot shows the median (central line), upper and lower quartiles (box limits), and min to max range (whiskers) by a two-tailed paired t-test. Source data are provided as a Source Data file.

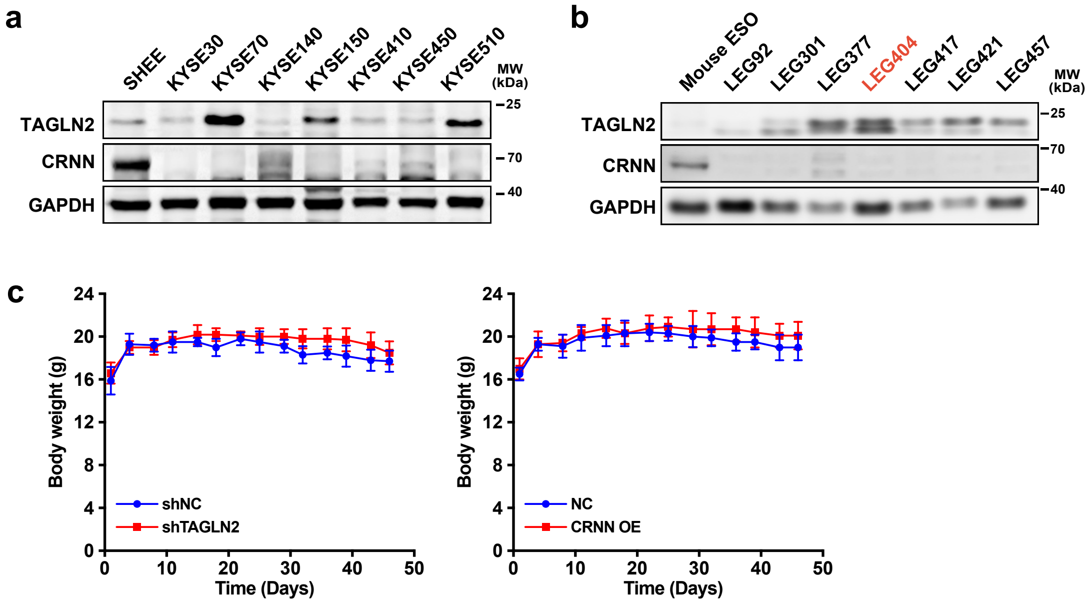

Supplementary Fig.7 Investigation of candidate genes' function in ESCC. a. Protein level of TAGLN2 and CRNN in normal esophageal cell line and ESCC cell lines. b. TAGLN2, and CRNN protein expression in different PDX cases analyzed by western blot. Mouse ESO is the abbreviation of Mouse esophagus. c. PDX mice (n = 6 mice) body weight was measured twice a week. Data are presented as mean values ± SD. Source data are provided as a Source Data file.

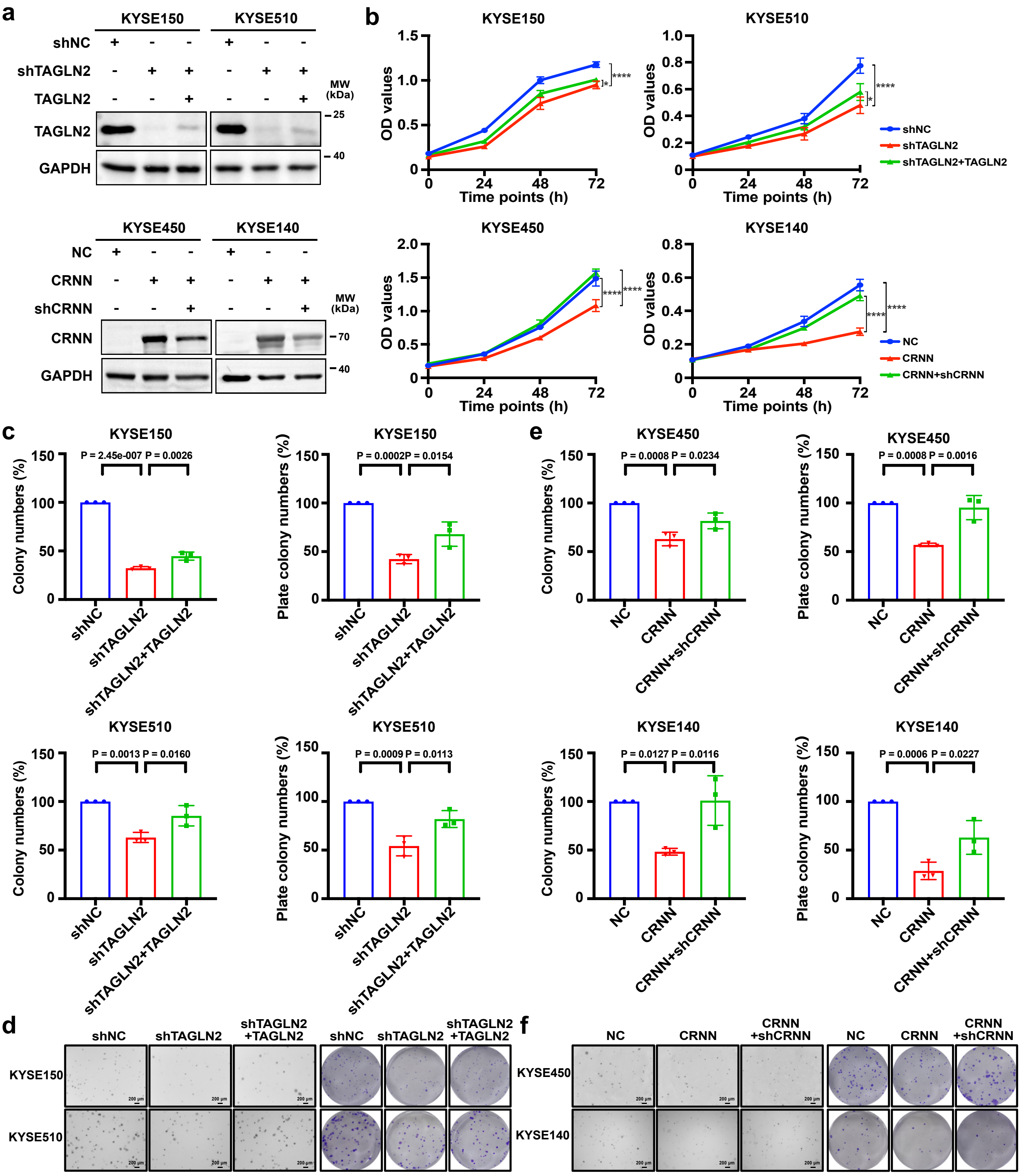

Supplementary Fig.8 Investigation of candidate genes' function by rescuing TAGLN2 and CRNN. a. The rescue efficiency was detected by Western blot. b. Cell proliferation analysis of rescuing TAGLN2 through overexpression and inhibiting CRNN through shRNA by MTT assay (n = 6 biological replicates by one-way ANOVA analysis, mean ± SD). **** p = 1.61e-008, **** p =2.03e-006 (shNC and shTAGLN2), * p = 0.0215, * p = 0.0441 (shTAGLN2 and shTAGLN2+TAGLN2) in KYSE150 and KYSE510, respectively.

**** p =2.57e-006, **** p = 1.11e-010 (NC and CRNN), **** p = 2.07e-007, **** p = 4.70e-009 (CRNN and CRNN+shCRNN) in KYSE450 and KYSE140, respectively. c. The colony formation ability of rescued *TAGLN2* was assessed by anchorage-independent cell growth assay and clonogenic formation assay. The data are shown as percentages compared with the control group. Statistical significance was analyzed using one-way ANOVA (n = 3, independent experiments, mean ± SD). d. Representative images of anchorage-independent cell growth assay and clonogenic formation assay of rescued *TAGLN2* (Scale bar, 200 μm). e. The colony formation ability of rescued *CRNN* was assessed by anchorage-independent cell growth assay and clonogenic formation assay. Statistical significance was analyzed by one-way ANOVA from n = 3 independent experiments (mean ± SD). f. Representative images of anchorage-independent cell growth assay and clonogenic formation assay of rescued *CRNN*. Source data are provided as a Source Data file.

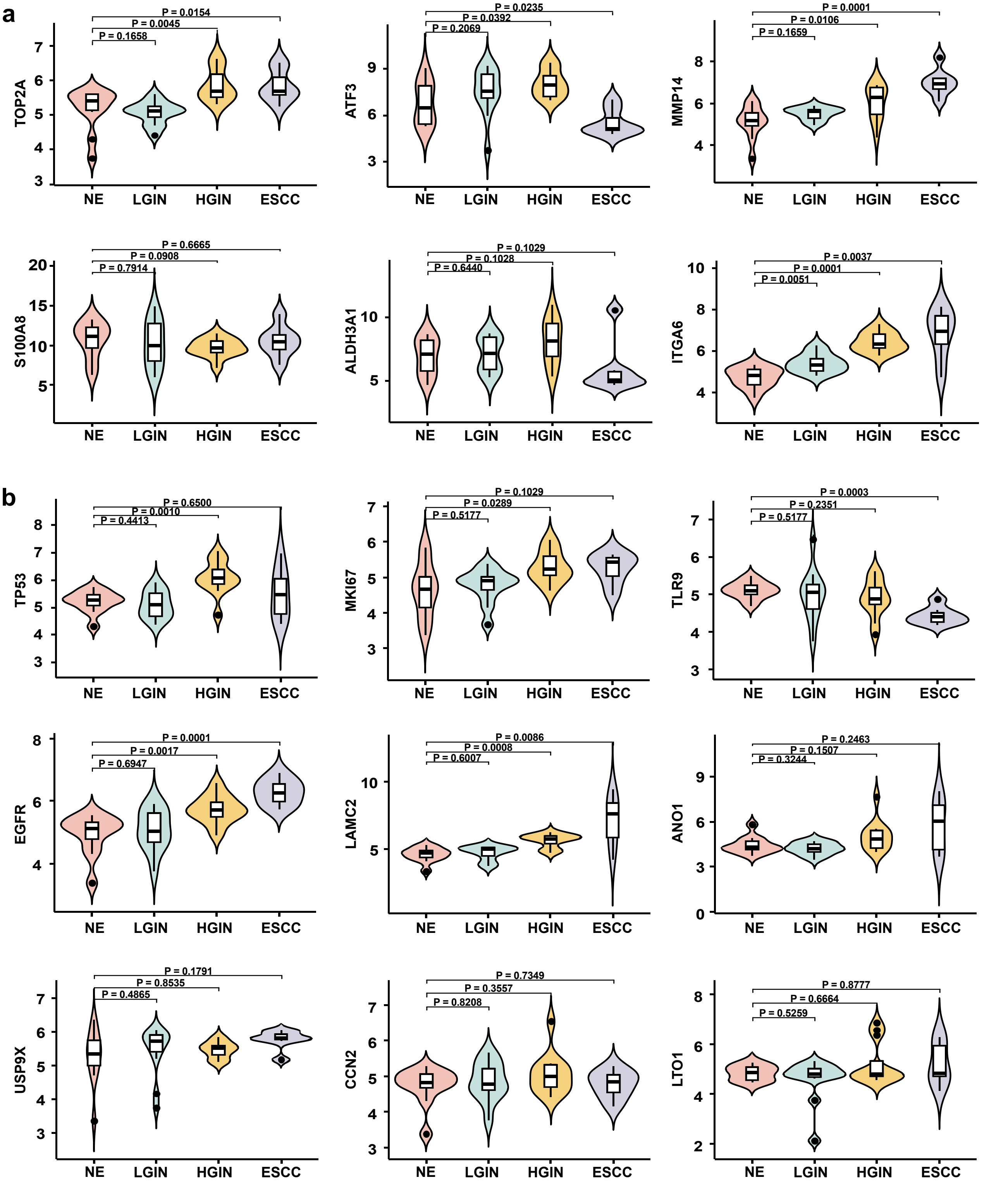

Supplementary Fig.9 Expression feature analysis of reported indicators for ESPL diagnosis. a. Key transitional genes reported from scRNA sequencing using continuous tumorigenic lesions of mice. b. Indicators that have been used in hospitals and preclinical studies. The mRNA expression of these mentioned genes was analyzed using the data from the spatial WTA analysis of this study. NE, LGIN, HGIN, and ESCC ROIs number (n = 11, 12, 12, 7). Mann-Whitney U test was used for the comparison with NE group. In the box

plots (a, b), the boxplot shows the median (central line), upper and lower quartiles (box limits), and 1.5 × interquartile range (whiskers).

Supplementary Table 1. Score definition and the relevant genes

Score name	Gene panel
Differentiation & Keratinization (D & K) score	*CRNN, MAL, EVPL, KLK12, KRT13, KRT4, PPL, SPINK5, SPRR1A, SPRR3*
Cancerization score	*KRT16, TAGLN2, IFI6, KRT10, KRT17, SPRR3, ANO1, KRT14, MRPL21, MYO1B*

Supplementary Table 2. Gene panel of knn algorithm

Gene group	Gene list
1	*CRCT1, CRISP3, CRNN, CYSRT1, EPS8L1*
6	*FAM3D, KRT78, MAL, PADI1, PRSS27*
11	*SLURP1, SPINK5, TMPRSS11B, COX6C, GPNMB*
16	*KRT16, KRT17, RPN2, TAGLN2, UQCRHL*

K = 6, n = 20
