# Supplementary material for: Spatial transcriptomics analysis of esophageal squamous precancerous lesions and their progression to esophageal cancer
Source: Nat Commun. 2023 Aug 8;14:4779. doi: 10.1038/s41467-023-40343-5 (PMC10409784; doi:10.1038/s41467-023-40343-5)
Supplement: Supplementary file 3 — Reporting Summary [file 41467_2023_40343_MOESM3_ESM.pdf]

nature portfolio

Corresponding author(s): Zigang Dong and Xiang Li

Last updated by author(s): Jul 22, 2023

Reporting Summary

Nature Portfolio wishes to improve the reproducibility of the work that we publish. This form provides structure for consistency and transparency in reporting. For further information on Nature Portfolio policies, see our Editorial Policies and the Editorial Policy Checklist.

Statistics

For all statistical analyses, confirm that the following items are present in the figure legend, table legend, main text, or Methods section.

n/a	Confirmed	
☐	☒	The exact sample size (*n*) for each experimental group/condition, given as a discrete number and unit of measurement
☐	☒	A statement on whether measurements were taken from distinct samples or whether the same sample was measured repeatedly
☐	☒	The statistical test(s) used AND whether they are one- or two-sided<br>*Only common tests should be described solely by name; describe more complex techniques in the Methods section.*
☒	☐	A description of all covariates tested
☐	☒	A description of any assumptions or corrections, such as tests of normality and adjustment for multiple comparisons
☐	☒	A full description of the statistical parameters including central tendency (e.g. means) or other basic estimates (e.g. regression coefficient) AND variation (e.g. standard deviation) or associated estimates of uncertainty (e.g. confidence intervals)
☐	☒	For null hypothesis testing, the test statistic (e.g. *F*, *t*, *r*) with confidence intervals, effect sizes, degrees of freedom and *P* value noted<br>*Give P values as exact values whenever suitable.*
☒	☐	For Bayesian analysis, information on the choice of priors and Markov chain Monte Carlo settings
☒	☐	For hierarchical and complex designs, identification of the appropriate level for tests and full reporting of outcomes
☒	☐	Estimates of effect sizes (e.g. Cohen's *d*, Pearson's *r*), indicating how they were calculated

Our web collection on statistics for biologists contains articles on many of the points above.

Software and code

Policy information about availability of computer code

Data collection	Digital count conversion
Data analysis	GraphPad Prism (Version 9.5.0), Image J (version 1.53k), R (version 4.2.1), GeoMx NGSPipeline (Version 2.0.0.16), GeoMx DSP Control Center (V.2.4.2.2), SCORPIUS (version 1.0.8), Spatialdecon safeTME (Version 1.8.0), Cibersort (Version 1.04), pheatmap package (Version 1.0.12), FactoMine package (Version 2.8), factoextra package (Version 1.0.7), ggplot2 package (Version 3.4.2), clusterProfiler package (Version 4.6.2), mlr (Version 2.19.1), digital image viewing software (version 12.3.2.8013). Code availability: The codes for data analysis used in this study are available at https://github.com/hrcnlab/escc_pipline.

For manuscripts utilizing custom algorithms or software that are central to the research but not yet described in published literature, software must be made available to editors and reviewers. We strongly encourage code deposition in a community repository (e.g. GitHub). See the Nature Portfolio guidelines for submitting code & software for further information.

Data

Policy information about availability of data

All manuscripts must include a data availability statement. This statement should provide the following information, where applicable:

- Accession codes, unique identifiers, or web links for publicly available datasets
- A description of any restrictions on data availability
- For clinical datasets or third party data, please ensure that the statement adheres to our policy

The raw sequence data generated in this study have been deposited in the GSA-Human database under accession code HRA003627 [https://ngdc.cncb.ac.cn/gsa-human]. The raw sequence data are available under restricted access for research purposes only, access can be obtained by the DAC (Data Access Committees) of the GSA-human database. According to the guidelines of GSA-human, all non-profit researchers are allowed access to the data, and the Principle Investigator of any research group is allowed to apply for Controlled-access of the data. The user can register the GSA database (https://ngdc.cncb.ac.cn/gsa-human/) and request the data. The approximate response time for accession requests is about 3 days. The access authority can be obtained for Research Use Only. The user can also contact the corresponding author directly. Once access has been approved, the data will be available to download for 2 months.
TCGA and GTEx database: https://xenabrowser.net
GEO data set: https://www.ncbi.nlm.nih.gov/geo/query/acc.cgi?acc=GSE161533; https://www.ncbi.nlm.nih.gov/geo/query/acc.cgi?acc=GSE159929; https://www.ncbi.nlm.nih.gov/geo/query/acc.cgi?acc=GSE160269
http://www.kmplot.com
Source data are provided with this paper.
The remaining data are available within the Article, Supplementary Information or Source Data files.

Human research participants

Policy information about studies involving human research participants and Sex and Gender in Research.

Reporting on sex and gender	This study has no sex- and gender-based analyses, and we do not collect this information.
Population characteristics	The tissue samples used in this study are the esophageal tissue samples from patients diagnosed as low-grade intraepithelial neoplasia (n = 6), high-grade intraepithelial neoplasia(n = 6), and esophageal squamous cell carcinoma (n = 7). Age, gender, lifestyle habits, and underlying medical conditions were not taken into consideration in this study.
Recruitment	The formalin-fixed paraffin-embedded (FFPE) tissue samples from patients diagnosed as low-grade intraepithelial neoplasia, high-grade intraepithelial neoplasia and esophageal squamous cell carcinoma were collected. The participants were selected based on the pathological diagnosis results and the H & E staining of tissue, without considering factors such as age or gender. No biases are involved in the participants selection.
Ethics oversight	This study were approved by Ethics Committee of Affiliated Cancer Hospital of Zhengzhou University (Zhengzhou, China). Written informed consent was provided by each patient before any investigation was conducted.

Note that full information on the approval of the study protocol must also be provided in the manuscript.

Field-specific reporting

Please select the one below that is the best fit for your research. If you are not sure, read the appropriate sections before making your selection.

☒ Life sciences ☐ Behavioural & social sciences ☐ Ecological, evolutionary & environmental sciences

For a reference copy of the document with all sections, see nature.com/documents/nr-reporting-summary-flat.pdf

Life sciences study design

All studies must disclose on these points even when the disclosure is negative.

Sample size	No statistical methods are used to predetermine sample size before experiments, The exact sample size of experiments is listed in the methods section or in the figure legends. We used as many samples as we could obtain for all the experiment.
Data exclusions	No data were excluded.
Replication	Information provided in Figure legends.
Randomization	For cell and animal experiments, all samples were randomly allocated into experimental groups. The categorization of tissue samples into different groups in this study is primarily based on the results of pathological diagnosis.
Blinding	The investigator who performed WTA analysis was blinded to patient information and group allocation during data collection. During the data

Blinding	collection and analysis of in vivo and in vitro experiments involving different experimental groups, we were not blinded to the group allocations. This was necessary for performing imaging and dynamic analysis such as animal experiments, as knowledge of the assigned experimental group was crucial. For image analysis, the experimental images were not blinded during imaging, because we need to find the relevant ESPL or ESCC region when taking pictures.

Reporting for specific materials, systems and methods

We require information from authors about some types of materials, experimental systems and methods used in many studies. Here, indicate whether each material, system or method listed is relevant to your study. If you are not sure if a list item applies to your research, read the appropriate section before selecting a response.

Materials & experimental systems

n/a	Involved in the study
☐	☒ Antibodies
☐	☒ Eukaryotic cell lines
☒	☐ Palaeontology and archaeology
☐	☒ Animals and other organisms
☒	☐ Clinical data
☒	☐ Dual use research of concern

Methods

n/a	Involved in the study
☒	☐ ChIP-seq
☒	☐ Flow cytometry
☒	☐ MRI-based neuroimaging

Antibodies

Antibodies used	anti-KRT16 (Proteintech, 66802-1-Ig, Clone No. 2H4D8, dilution: 1:2000 for WB, 1:1000 for IHC)<br>anti-KRT17 (Proteintech, 17516-1-AP, dilution: 1:1000 for WB, 1:50 for IHC)<br>anti-TAGLN2 (Proteintech, 10234-2-AP, dilution: 1:1000 for WB, 1:100 for IHC)<br>anti-CRNN (Proteintech, 11799-1-AP, dilution: 1:1000 for WB, 1:200 for IHC)<br>anti-MAL (Invitrogen, MA5-32924, Clone No. B5-G3, dilution: 1:1000 for WB, 1:100 for IHC)<br>anti-CD68 (Abcam, ab955, Clone No. KP1, dilution: 1:50 for IF)<br>anti-alpha-SMA (Abcam, ab124964, Clone No. EPR5368, dilution: 1:200 for IF)<br>anti-GAPDH (ZSGB-BIO, TA-08, Clone No. OTI2D9, 1:2000 for WB)<br>Goat anti-rabbit IgG H&L (HRP) (Abcam, ab205718, 1:5000 for WB)
Validation	66802-1-Ig validated for FC, IF, IHC, WB, ELISA by manufacturer. https://www.ptgcn.com/products/Cytokeratin-16-Antibody-66802-1-Ig.htm#tested-applications.<br>17516-1-AP validated for FC, IF, IHC, WB, ELISA by manufacturer. https://www.ptgcn.com/products/KRT17-Specific-Antibody-17516-1-AP.htm#tested-applications.<br>10234-2-AP validated for IF, IHC, WB,ELISA by manufacturer. https://www.ptgcn.com/products/TAGLN2-Antibody-10234-2-AP.htm#tested-applications.<br>11799-1-AP validated for IHC, WB, ELISA by manufacturer. https://www.ptgcn.com/products/CRNN-Antibody-11799-1-AP.htm#tested-applications.<br>MA5-32924 validated for IHC, WB, Flow Cytometry by manufacturer. https://www.thermofisher.cn/cn/zh/antibody/product/MAL-Antibody-clone-B5-G3-Monoclonal/MA5-32924.<br>ab955 validated for ICC/IF, WB, IHC-P by manufacturer. https://www.abcam.com/products/primary-antibodies/cd68-antibody-kp1-ab955.html<br>ab124964 validated for ICC/IF, WB, IHC-P and Flow Cyt (Intra) by manufacturer. https://www.abcam.com/products/primary-antibodies/alpha-smooth-muscle-actin-antibody-epr5368-ab124964.html<br>TA-08 validated for WB. http://www.zsbio.com/product/TA-08.<br>ab205718 validated for IHC-P, WB, ELISA, IP by manufacturer. https://www.abcam.cn/products/secondary-antibodies/goat-rabbit-igg-hl-hrp-ab205718.html

Eukaryotic cell lines

Policy information about cell lines and Sex and Gender in Research

Cell line source(s)	KYSE150 cell line was obtained from Cell Bank of Chinese Academy of Sciences (cat: CBTCCCAS, Shanghai, China). KYSE140, KYSE450, KYSE510, KYSE30, KYSE70, and KYSE410 were preserved and donated by Professor Ziming Dong (the Department of Pathophysiology, school of basic medical sciences of Zhengzhou University). Professor Enmin Li (Shantou University) donated the normal human esophagus immortalized epithelial cell (SHEE).
Authentication	All the cell lines used in this study are not found in the cell lines registered as misidentified cell lines in the International Cell Line Authentication Committee (ICLAC). KYSE140, KYSE150, KYSE450, KYSE510, KYSE30, KYSE70, and KYSE410 cell lines were validated by STR analysis.
Mycoplasma contamination	The cell lines were tested negative for mycoplasma contamination.
Commonly misidentified lines<br>(See ICLAC register)	No such misidentified lines were used in the study.

Animals and other research organisms

Policy information about studies involving animals; ARRIVE guidelines recommended for reporting animal research, and Sex and Gender in Research

Laboratory animals	Eight-week CB17 SCID female immunodeficient mice were obtained from Cyagen Biosciences (Suzhou, Jiangsu, China). The housing conditions for mice included a 12-hour dark/light cycle, with an ambient temperature maintained at approximately 24 °C and a humidity level around 60%. The PDX tumors were transplanted to mice and the experiment started from the 7th day after transplant (showed as day 1). Tumor volume and mice body weight was measured twice a week and at the same time conducted intratumor injection of designated virus of shNC, shTAGLN2, NC and CRNN. Tumors were removed, weighted and photographed at the 46 days after euthanized the mice.
Wild animals	No wild animals were used in the study.
Reporting on sex	No specific consideration was given to mice sex.
Field-collected samples	No field-collected samples were used in the study.
Ethics oversight	This animal study was approved by the Ethics Committee of China-US (Henan) Hormel Cancer Institute (Zhengzhou, Henan, China).

Note that full information on the approval of the study protocol must also be provided in the manuscript.
